# Measuring quality of life with the Parkinson’s Disease Questionnaire-39 in people with cognitive impairment

**DOI:** 10.1371/journal.pone.0266140

**Published:** 2022-04-01

**Authors:** Aline Schönenberg, Tino Prell

**Affiliations:** 1 Department of Geriatrics, Halle University Hospital, Halle, Germany; 2 Department of Neurology, Jena University Hospital, Jena, Germany; 3 Center for Healthy Ageing, Jena University Hospital, Jena, Germany; Chinese Academy of Sciences, CHINA

## Abstract

**Introduction:**

Quality of life (QoL) is a key outcome in healthcare. However, whether cognitively impaired people with Parkinson’s disease (PD) can reliably self-report QoL is unclear, and patients are often excluded from studies based on cognition test scores. The aim of this analysis was to assess the validity of the Parkinson’s Disease Questionnaire-39 (PDQ-39) in PD patients with and without cognitive impairment.

**Methods:**

In this study, 221 individuals with PD completed the PDQ-39, Montreal Cognitive Assessment (MOCA), and Beck’s Depression Inventory (BDI-II). The PDQ-39’s internal consistency, convergent validity with BDI-II, and floor and ceiling effects were analyzed for patients with and without cognitive impairment.

**Results:**

Ninety-four patients showed cognitive impairment (MOCA <21), whereas 127 patients had mild/no impairment. Both MOCA groups differed significantly with regards to PD severity. The PDQ-39’s internal consistency was adequate for most subdomains in both MOCA groups, but floor effects were present for the subdomains Stigmatization, Social Support and Communication, regardless of impairment. For some subdomains, the PDQ-39’s convergent validity with the BDI receded in the low MOCA group but remained significant for most PDQ-39 domains, especially for the PDQ total score (r = .386, p < .001) and for the subdomain emotional well-being (r = .446, p < .001).

**Conclusion:**

The PDQ-39 can be used to measure QoL in cognitively impaired PD patients, thus test scores indicating cognitive impairment alone should not lead to exclusion of PD patients from clinical studies. Although the correlation between BDI-II and PDQ-39 shrinks for some subdomains in cognitively impairment patients, this finding may be explained by the difference in PD severity, as factors influencing QoL may shift with increasing age and PD symptoms.

## Introduction

Health-related quality of life (QoL) describes a patient’s interpretation of their current health and is a key outcome in healthcare, especially for chronic neurodegenerative disorders, including Parkinson’s disease (PD). Nonmotor symptoms have one of the greatest influences on QoL in patients with PD, with depression alone accounting for a large amount of the variability in QoL [1, 2]. Although different QoL instruments have been validated [2], whether cognitively impaired patients can reliably self-report QoL remains unclear. There are QoL instruments specifically constructed for cognitively impaired patients, however, the use of these instrument in both clinical and research settings is limited due to unavailability and difficulties regarding feasibility (e.g. costs, duration, scoring), or lack of psychometric characteristics [3]. Additionally, results may vary with severity of cognitive deficits [4, 5]. As PD is a progressive disease with characteristic symptoms, the use of a disease-specific QoL instrument is often reasonable and necessary [6]. Of those specific instruments, the PD Questionnaire-39 (PDQ-39) is most widely used [6, 7]. Given the high prevalence of cognitive deficits in PD [8, 9], it is important to assess whether PD patients with cognitive deficits can make reliable statements about their QoL using the PDQ-39. This is crucial for both health practice and research to ensure that patients are not unnecessarily excluded from clinical research based on cognitive impairment scores alone.

QoL ratings provided by relatives or caregivers do not capture the patients’ evaluations and rate QoL systematically lower than the patients themselves, and previous research suggests that patients can make reliable statements about QoL up into late stages of dementia [10]. Whether those differences between self-reports and proxy ratings stem from low reliability of patient or proxy ratings remains unclear [4]; thus, we decided to not compare self-reported QoL with proxy assessments. Instead, we assessed the validity of QoL assessments in patients with PD with and without cognitive impairment using the well-documented relationship between QoL and depression [1, 2].

For this purpose, internal consistency of the PDQ-39 was assessed for patients with and without cognitive impairment, and convergent validity was examined with depression questionnaires.

## Methods

### Participants and assessments

This manuscript provides an additional analysis of an existing dataset, thus details on the data collection procedure and demographic and clinical data regarding PD severity are given elsewhere [11]. This study was approved by the Ethics Committee of Jena University Hospital and conducted according to the Declaration of Helsinki. A total of 230 inpatients with PD were recruited from January 2019 to January 2020 from the Department of Neurology, Jena University Hospital, Germany. Inclusion criteria consisted of PD as a primary diagnosis as well as absence of severe dementia and delirium. Because of missing data in the measures used for this additional manuscript, nine patients were excluded from the analysis, leaving 221 datasets. Since there are no sample size guidelines for content validation and sample sizes vary across the literature, our estimation was based on the recommended sample size of a minimum of 100 patients for construct validation studies, with recommendations varying between 100 and 250 [12].

All patients or legal representatives provided written informed consent. Data were collected by trained research staff, and tests were performed at the hospital during medication on-phase. PD diagnosis was made by a trained neurologist according to the Movement Disorder Society (MDS) criteria. Cognition was assessed using the Montreal Cognitive Assessment (MOCA) [13] in face-to-face testing, enabling us to gather an impression of each patient’s ability to understand and complete a questionnaire. Therefore, we included patients with MOCA scores below the threshold of 21 points for PD dementia (PDD) [14] if they could answer the questions coherently. Accordingly, the cohort was split into two groups: low MOCA (<21 points) and high MOCA (≥21 points). For a more refined analysis, the cohort was additionally split into three groups (MOCA <21, MOCA 21–25, MOCA ≥26) to confirm the results.

QoL was assessed using the PDQ-39, a self-report questionnaire depicting the frequency of impairments on a 4–point Likert scale ranging from “Never” to “Always”. The PDQ-39 can be summarized in a total score as well as eight subdomains regarding mobility, activities of daily living (ADL), emotional well-being, stigmatization, social support, cognition, communication, and bodily discomfort, with higher scores indicating more frequent impairment in these domains [7].

Beck’s Depression Inventory-II (BDI-II) was used to assess depression. The BDI-II assesses the severity of depressive symptoms across 21 self-report items cumulating in an overall sum score, with higher scores indicating higher severity [15].

Additionally, the non-motor symptom questionnaire (NMS-Q) [16] was used to confirm the results of the comparison with the BDI. Of note, although the NMS-Q includes questions regarding mental well-being, it assesses a wide range of non-motor symptoms and is not focused on mental well-being, thus the results are reported in the supplementary materials as an additional indicator. Physical functioning was assessed with the Movement Disorder Society MDS-Unified Parkinson’s Disease Rating Scale (UPDRS), an assessment performed by trained medical staff evaluating the severity of common nonmotor and motor symptoms of PD [17]. Again, higher scores indicate more severe symptoms.

### Statistical analysis

Statistical analyses were performed using Statistical Package for the Social Sciences (version 27.0; IBM Corp., Armonk, NY, USA) and R (version 4.1.1; R Foundation for Statistical Computing, Vienna, Austria). *P-*values below 0.05 denote statistical significance.

Initially, the cohort was analyzed using descriptive statistics (mean, ± standard deviation (SD), and percentages), and normal distribution was assessed with the Shapiro–Wilk test. Group comparisons were performed with Mann–Whitney U test for metric variables using the R-Package *rstatix* [18], and the chi-square test for categorical variables. The 95% confidence intervals and effect sizes are given where applicable. Effect sizes for group comparisons (two-sample rank-sum tests) are calculated by dividing the z statistic by the square root of the sample size and can be interpreted as small effects (0.10 - < 0.3), moderate effects (0.30 - < 0.5) and large effects (> = 0.5) [18].

To assess the reliability of the PDQ-39, scores and internal consistency were assessed for both MOCA groups. Floor and ceiling effects describe the proportion of patients reaching the highest (ceiling) or lowest (floor) possible score and were considered present if at least 15% of the respondents reached this respective score. Internal consistency was measured using Cronbach’s alpha and considered adequate for values higher than 0.70 [19]. Convergent validity was assessed with the Spearman correlation of all PDQ-39 domains with the BDI-II. Recommendations indicate that correlations between instruments measuring similar constructs should be greater than or equal to 0.5, thus a correlation of 0.5 was expected between the BDI-II and the PDQ-39 subscale emotional well-being. For instruments measuring similar but not identical constructs, correlation should lie between 0.3 and 0.5. Correlations of 0.1, 0.3, and 0.5 were considered weak, moderate, and strong correlations, respectively [19].

## Results

The cohort (N = 221) included 89 (40.3%) female and 132 (59.7%) male PD patients between the age of 40 and 89 (mean 70.81 ± 8.32) years. Ninety-four patients (42.5%) had low MOCA scores (<21 points), and 127 patients (57.5%) had high MOCA scores (≥21 points) (S1 Fig). Detailed clinical and demographic data are shown in Table 1.

**Table 1:** Clinical and demographic data of people with low and high MOCA scores.

	**MOCA < 21**				**MOCA ≥ 21**					
		**Count (%)**			**Count (%)**				***p***	***Φ***
Sex	female	42 (44.7)			47 (37.0)				.312	.08
	male	52 (55.3)			80 (63.0)					
Education level	low	26 (32.9)			15 (12.9)				.003	.250
	middle	21 (26.6)			46 (39.7)					
	high	32 (40.1)			55 (47.4)					
		**Mean (SD)**		**Median (IQR)**	**Mean (SD)**	**Median (IQR)**			***p***	***r***
Age (y)		73.80 (8.12)		75 (9)	68.60 (7.80)	69 (10)			< .001	.350
Disease duration (y)		7.96 (5.76)		7 (7)	7.30 (5.37)	6 (8)			0.405	.056
Hoehn and Yahr				3 (1)				3 (1)	.001	.218
MDS-UPDRS		76.20 (27.60)		(42)	62.10 (28.20)	62 (39)			.013	.237
NMS-Quest		12.10 (4.82)		12 (8)	9.90 (5.00)	9 (7)			.001	.223
BDI-II		14.30 (7.72)		13 (9)	12.2 (8.87)	10 (9)			.005	.186
MOCA		17. 29 (2.98)		18 (3)	24.20 (2.36)	24 (4)			< .001	.857
PDQ-39		34.60 (15.80)		35 (23)	27.40 (16.90)	27 (26)			.001	.225
Mobility		48.76 (26.70)		48 (37.5)	37.88 (27.59)	31 (48)			.004	.192
ADL		41.48 (25.23)		42 (44.8)	30.90 (25.28)	25 (38)			.001	.217
EWB		36.84 (20.80)		38 (28)	28.09 (22.51)	25 (38)			.002	.207
Stigmatization		21.87 (19.72)		19 (32)	20.02 (22.18)	12 (31)			.214	.084
Social Support		21.62 (22.36)		17 (33)	13.02 (18.76)	0 (17)			.001	.209
Cognition		36.50 (29.32)		38 (25)	30.08 (22.67)	25 (32)			.011	.171
Communi-cation		30.65 (21.85)		33 (25)	25.13 (23.83)	17 (42)			.047	.134
Bodily Discomfort		38.85 (22.61)		42 (25)	34.82 (24.48)	33 (33)			.164	.094

MDS-UPDRS: MDS-sponsored revision of the unified PD rating scale, NMS-Quest: Nonmotor-Symptoms Questionnaire, BDI II = Beck’s Depression Inventory, PDQ-39: Parkinson’s Disease Questionnaire 39, MOCA: Montreal Cognitive Assessment. p depicts significant difference between mean scores for each domain based on Mann-Whitney U test or Chi^2^ test, r depicts the effect size of this comparison based on two-sample rank-sum test, *Φ = Phi*.

Significant differences in BDI-II score (*p* = 0.005), PDQ-39 total score (*p* = 0.001), age (*p* < 0.001), HY stages (*p* = 0.001), NMS-Q score (*p* = 0.001), and MDS-UPDRS score (*p* = 0.013) were observed between the two MOCA groups (Table 1). Considering the PDQ-39 subdomains, patients in the low MOCA group scored worse in mobility (*p* = 0.004), activities of daily living (*p* = 0.001), emotional well-being (*p* = 0.002), social support (*p* = 0.002), cognition (*p* = 0.011), and communication (*p* = 0.047) (see also S2 Fig).

Floor effects were present for the PDQ-39 subdomains stigmatization, social support, and communication for both MOCA groups. Internal consistency was adequate and comparable between groups for most PDQ-39 subdomains (Table 2).

**Table 2:** Parkinson’s Disease Questionnaire-39 scores and internal consistency in people with low and high Montreal Cognitive Assessment scores.

PDQ-39 Scale	MOCA <21			MOCA ≥21		
	Floor	Ceiling	α	Floor	Ceiling	α
PDQ-39 sum	0	0		0	0	
Mobility	5.3	0	0.927	4.7	1.6	0.942
Activities of daily living	7.4	0	0.882	6.3	0.8	0.898
Emotional well-being	3.2	0	0.864	12.6	0.8	0.900
Stigmatization	22.3	0	0.775	31.5	0	0.823
Social support	34.0	0	0.70	51.2	0	0.681
Cognition	7.4	0	0.737	10.2	0.8	0.783
Communication	18.1	0	0.719	23.8	0.8	0.794
Bodily discomfort	8.5	0	0.637	12.6	0	0.675

PDQ-39: Parkinson’s Disease Questionnaire-39; BDI-II: Beck’s Depression Inventory-II, MOCA: Montreal Cognitive Assessment.; α = Cronbach’s Alpha.

The patients in both MOCA groups responded comparably to the BDI-II items. A significant difference was only found in the BDI-II items 1: sadness (*p* = 0.011), 4: loss of pleasure (*p* = .008), 16: change in Sleeping Habits (*p* = .04), and 19: difficulties concentrating (*p* = .008) however, effect sizes were small (all r < .18), indicating that the groups did not differ substantially (S1 Table).

To estimate the convergent validity of the PDQ-39, we correlated each subdomain to the BDI-II total score (Table 3). In the high MOCA group, all PDQ-39 subdomains and the total score showed moderate to high correlations with the BDI-II. For patients with low MOCA scores, correlations remained comparable, although they were slightly lower for some subdomains. However, the PDQ-39 total score and all subdomains, except for stigmatization and bodily discomfort, continued to show moderate, significant correlation with the BDI-II.

**Table 3:** Convergent validity of the Parkinson’s Disease Questionnaire-39 in people with low and high Montreal Cognitive Assessment scores.

PDQ-39	MOCA <21			MOCA ≥ 21		
	BDI-II			BDI-II		
	Spearman	*P*	95% CI	Spearman	*P*	95% CI
PDQ-39 total score	0.386	<0.001	.199, .546	0.50	<0.001	.39, .64
Mobility	0.265	.01	.065, .444	.281	.001	.113, .434
Activities of daily living	.250	.015	.050, .430	.331	<0.001	.166, .477
Emotional well-being	0.446	<0.001	.267, .595	0.634	<0.001	.517, .728
Stigmatization	0.133	0.203	-.072, .326	0.257	0.004	.086, .413
Social support	0.400	<0.001	.215, .558	.282	0.001	.114, .435
Cognition	0.274	0.008	.076, .452	0.613	<0.001	.491, .711
Communication	0.238	0.021	.037, .423	0.244	.006	.073, .401
Bodily discomfort	0.158	0.129	-.046, .349	0.295	0.001	.128, .447

PDQ-39, Parkinson’s Disease Questionnaire-39; BDI-II, Beck’s Depression Inventory-II; MOCA, Montreal Cognitive Assessment.

The strongest correlations were found between the BDI-II and the emotional well-being subdomains in both groups (Table 3). The main findings did not change when splitting the cohort into three groups (low MOCA, <21; mild cognitive impairment, 21–25; normal, >25), and when comparing the PDQ-39 to the NMS-Q or the UPDRS (S2–S4 Tables). For the comparison with the UPDRS, the subscales mobility, ADL and Bodily Discomfort were chosen as an additional assessment of the self-report of physical problems impacting QoL for both MOCA groups where available.

## Discussion

We conducted this study to assess the accuracy of the PDQ-39 in PD patients with low and high MOCA scores and examine whether cognitively impaired individuals with PD can make reliable self-report statements about their QoL. Certain instruments have been validated to assess cognitively impaired persons, but they are not widely used and results may vary across cohorts and instruments [2, 4]. Proxy ratings of QoL for older adults are not reliable sources of information on the patients’ QoL assessments [4] and validity assessments of instruments may vary depending on cohort factors and choice of comparison instruments [2]. Thus, there is no gold standard QoL instrument for convergent validity assessments.

Overall, our results indicate that the responses to the PDQ-39 are reliable for PD patients with lower MOCA scores in most PDQ-39 subdomains. Of note, our lowest MOCA score was 12 points. Therefore, we cannot make statements about the accuracy below this threshold. As expected, we observed moderate to strong associations between depression and PDQ-39 subdomains cognition and emotional well-being subdomains, which are both primarily related to mood [1, 2, 20]. The subdomains stigmatization, communication, and social support showed floor effects; and as confirmed by other studies, the social support and bodily discomfort subdomains also had below adequate internal consistency, indicating that some subdomains may not have been appropriate in both MOCA groups [7, 21]. For this reason, Cronbach’s Alpha for those domains should be interpreted with caution. However, it is neither the intention nor in the scope of this analysis to judge the adequacy of the PDQ. In addition, internal consistency and floor effects were comparable between PD patients with high and low MOCA scores, indicating that cognitive impairment was not responsible for these responses.

Although convergent validity remained comparable for most subdomains in the low MOCA group, some subdomains showed changes compared to the high MOCA group, indicating that the association between the instruments shifts with increasing cognitive impairment. This seems reasonable as, comparable to other studies [22, 23], the PD patients in the low MOCA group were older and scored worse in HY stages, MDS-UPDRS, and NMS-Q, which all have an additional influence on QoL. Thus, the changing association between PDQ-39 and BDI-II in the low MOCA group may be influenced by shifting QoL due to advanced age and disease progression that may not be fully captured by all instruments [1, 2]. The BDI-II and PDQ-39 do not aim to measure the exact same constructs, and although the BDI-II can capture certain aspects of QoL [15] as mood plays a pivotal role [1, 2], it is intended that the PDQ-39 encompasses symptoms not registered by the BDI-II. Emotional well-being and cognition, two PDQ-39 subdomains related primarily to mood [20], show significant correlation with the BDI-II even in the low MOCA group, whereas other domains not primarily assessed by the BDI-II changed in correlation, suggesting that the impact of those symptoms exceeds the scope of the BDI-II at a certain severity stage.

Regarding the characteristics of the described cohort, we thus considered the correlation between BDI-II and PDQ-39 scores in the low MOCA group to be expected. As most subdomains still show comparable internal consistency and appropriate convergent validity for instruments measuring similar but not identical constructs [19], we conclude that the PD patients in our cohort with MOCA scores below the cutoff of 21 for PDD can reliably self-report QoL using the PDQ-39. As the BDI-II does not capture all aspects of QoL and cannot encompass all PDQ-39 subdomains, further studies are needed to validate the QoL assessment of cognitively impaired individuals using other QoL instruments. Overall, we conclude that a low score in cognitive impairment measures alone should not be a reason to exclude PD patients from clinical studies on QoL.

This study is not without limitations. The cross-sectional design does not allow for interpretations of causality, and the sample of PD patients restricts generalization across other cohorts, not allowing any conclusions for overall QoL assessment in persons with cognitive impairment not suffering from PD. Lastly, although we included patients with MOCA scores below the cutoff for PDD, we did not include patients with severely impaired cognition, as filling in a questionnaire is impossible in such cases, but see [23] for an assessment of QoL in patients with PDD. Notably, the MOCA alone cannot replace a comprehensive neuropsychological assessment of cognition and does not represent an actual diagnosis of cognitive impairment; however, the MOCA or comparable measures are often used in clinical studies to exclude patients below a certain cut-off, leading to an underrepresentation of these patients and their needs in clinical studies. Although a first statement can be made that these PD patients should not be excluded solely on the basis of such MOCA scores, more studies are needed to elucidate the measurement of QoL in patients with varying degrees of cognitive impairment, e.g. assessing test-retest reliability or utilizing several QoL instruments for comparison. Another promising route to assessing the usability of the PDQ-39 in PD patients with cognitive impairment is its strong relationship with anxiety, as anxiety is also highly prevalent in PD and just as debilitating for QoL [2, 24]. Thus, in future studies, similar analyses should be performed using anxiety as another measure for convergent validity. Additionally, more research is needed to understand the use of QoL measures in cognitively impaired patients without PD.

## Supporting information

S1 Fig Frequency of Montreal cognitive assessment (MOCA) total scores. (DOCX)

S2 Fig PDQ-39 responses for persons with low and high MOCA (mean with standard deviation). (DOCX)

S1 Table BDI Responses for persons with low and high MOCA. (DOCX)

S2 Table Convergent validity of the PDQ-39 for people with varying levels of cognitive impairment. (DOCX)

S3 Table Convergent validity of the PDQ-39 and the NMS-Q for people with low and high MOCA score. (DOCX)

S4 Table Convergent validity of the PDQ-39 and the UPDRS for people with low and high MOCA score. (DOCX)
