# Supplementary figures and images for: Measuring quality of life with the Parkinson’s Disease Questionnaire-39 in people with cognitive impairment
Source: PLoS One. 2022 Apr 1;17(4):e0266140. doi: 10.1371/journal.pone.0266140 (PMC8975160; doi:10.1371/journal.pone.0266140)

**Supplement Figure 1**. Frequency of Montreal cognitive assessment (MOCA) total scores.


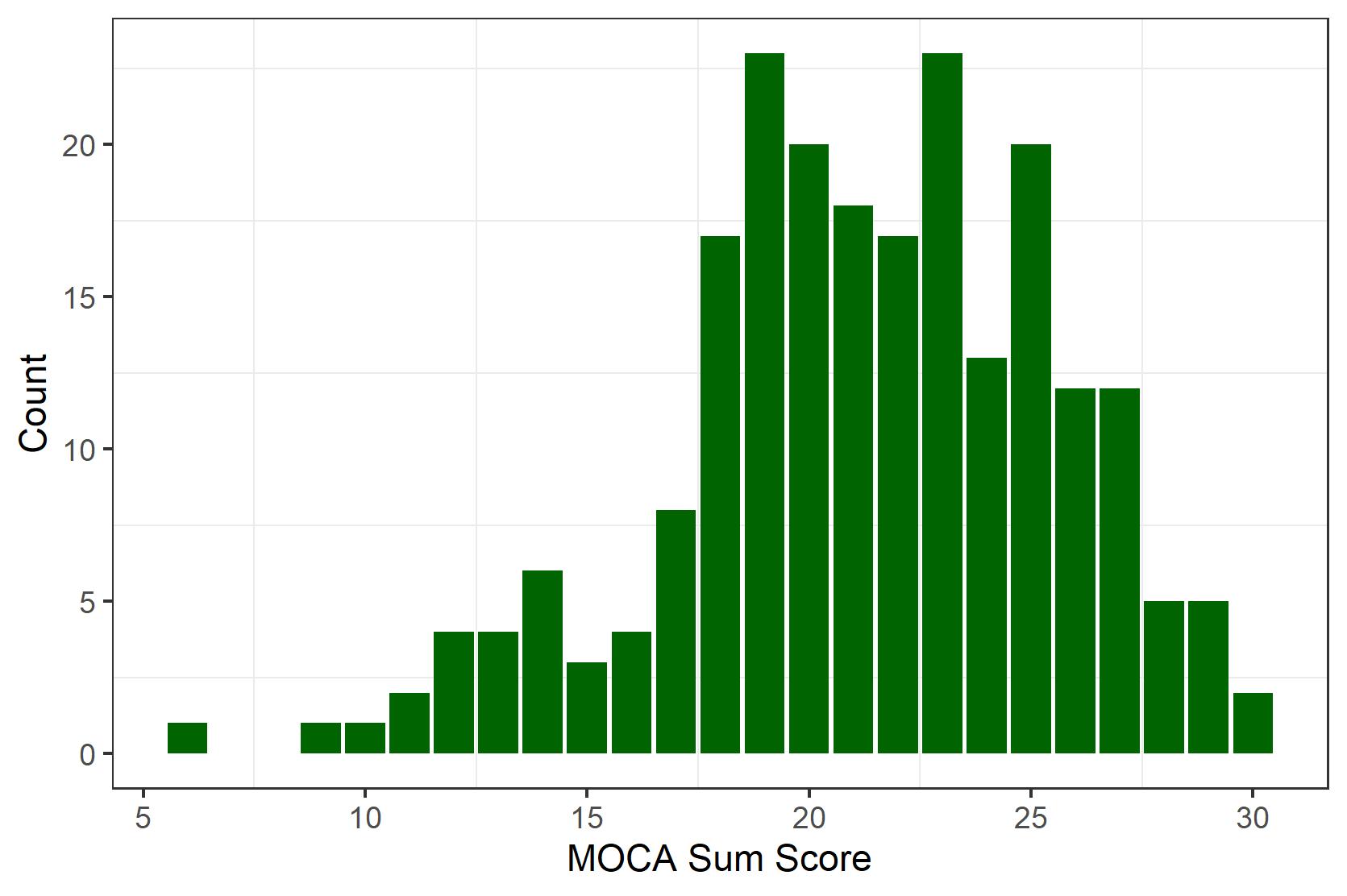

Supplement: S1 Fig — (DOCX) [file pone.0266140.s001.docx]
