# Supplementary material for: Measuring quality of life with the Parkinson’s Disease Questionnaire-39 in people with cognitive impairment
Source: PLoS One. 2022 Apr 1;17(4):e0266140. doi: 10.1371/journal.pone.0266140 (PMC8975160; doi:10.1371/journal.pone.0266140)
Supplement: S2 Fig — (DOCX) [file pone.0266140.s002.docx]

**Supplement Figure 2.** PDQ-39 responses for persons with low and high MOCA (mean with standard deviation)


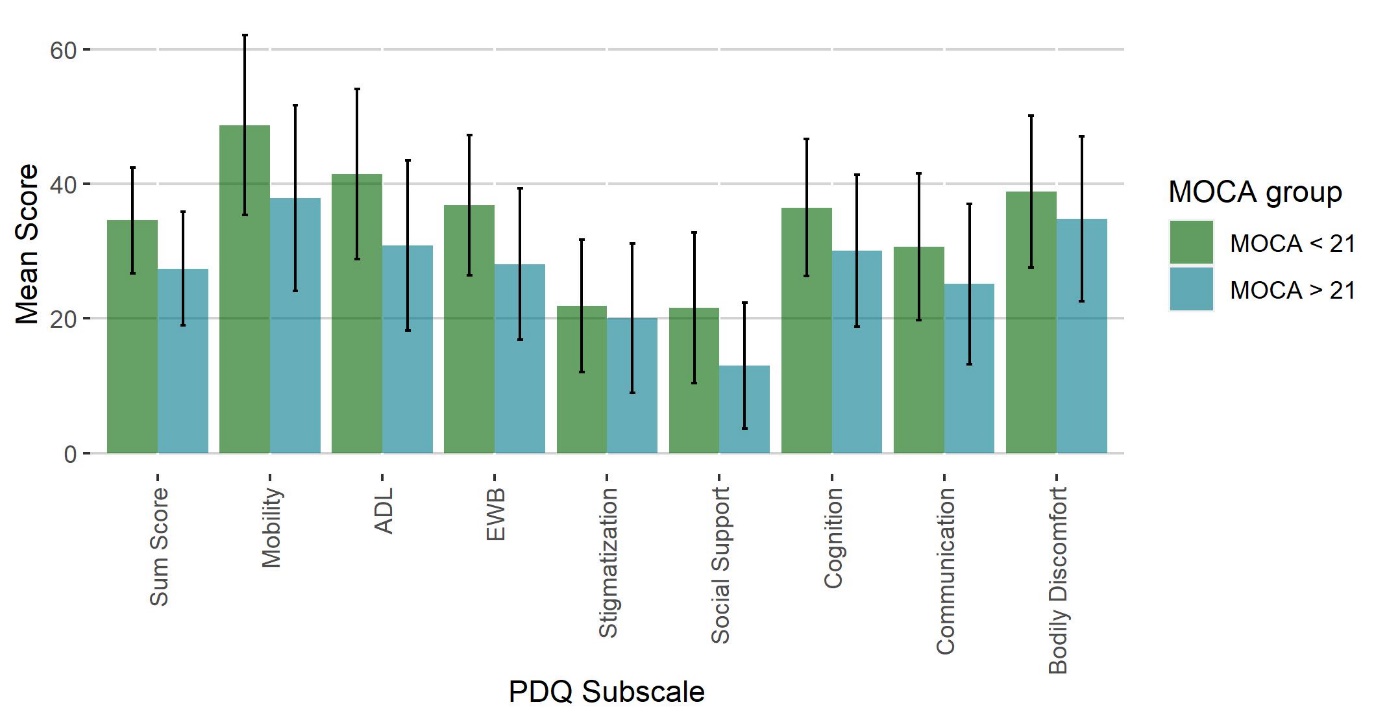
Note: ADL = Activities of Daily Living, EWB = Emotional Wellbeing
