# Supplementary material for: Measuring quality of life with the Parkinson’s Disease Questionnaire-39 in people with cognitive impairment
Source: PLoS One. 2022 Apr 1;17(4):e0266140. doi: 10.1371/journal.pone.0266140 (PMC8975160; doi:10.1371/journal.pone.0266140)
Supplement: S1 Table — (DOCX) [file pone.0266140.s003.docx]

**Supplement Table 1.** BDI Responses for persons with low and high MOCA.

| **BDI Item** | **MOCA < 21** | | | | **MOCA** ≥ **21** | | | | **Group Comparison** | |
| --- | --- | --- | --- | --- | --- | --- | --- | --- | --- | --- |
|  | **Mean** | **SD** | **Mdn** | **IQR** | **Mean** | **SD** | **Mdn** | **IQR** | **p** | **r** |
| 1 Sadness | .57 | .60 | 1 | 1 | .38 | .52 | 0 | 1 | **.011** | **.171** |
| 2 Pessimism | .61 | .85 | 0 | 1 | .60 | .81 | 0 | 1 | .953 | .004 |
| 3 Failure | .32 | .61 | 0 | 0 | .32 | .64 | 0 | 0 | .818 | .016 |
| 4 Loss of Pleasure | 1.07 | .76 | 1 | 0 | .80 | .68 | 1 | 1 | **.008** | **.178** |
| 5 Feeling of Guilt | .25 | .46 | 0 | 0 | .23 | .51 | 0 | 0 | .496 | .046 |
| 6 Feeling of Punishment | .27 | .69 | 0 | 0 | .27 | .71 | 0 | 0 | .937 | .005 |
| 7 Disconformity with oneself | .27 | .63 | 0 | 0 | .20 | .46 | 0 | 0 | .753 | .021 |
| 8 Self-criticism | .43 | .63 | 0 | 1 | .37 | .66 | 0 | 1 | .460 | .050 |
| 9 Suicidal thoughts | .16 | .40 | 0 | 0 | .18 | .43 | 0 | 0 | .734 | .023 |
| 10 Crying | .55 | .81 | 0 | 1 | .53 | .91 | 0 | 1 | .388 | .058 |
| 11 Agitation | .86 | .91 | 1 | 1 | .79 | .87 | 1 | 1 | .565 | .039 |
| 12 Loss of interest | .60 | .94 | 0 | 1 | .48 | .64 | 0 | 1 | .914 | .007 |
| 13 Indecision | .71 | .68 | 1 | 1 | .69 | .75 | 1 | 1 | .554 | .040 |
| 14 Devaluation | .52 | .71 | 0 | 1 | .42 | .67 | 0 | 1 | .214 | .084 |
| 15 Loss of energy | 1.12 | .75 | 1 | 1 | 1.06 | .65 | 1 | 0 | .507 | .045 |
| 16 Changes in Sleeping Habits | 1.30 | .89 | 1 | 1 | 1.06 | .84 | 1 | 2 | **.040** | **.138** |
| 17 Irritability | .52 | .67 | 0 | 1 | .43 | .61 | 0 | 1 | .326 | .066 |
| 18 Changes in Appetite | .73 | .88 | 1 | 1 | .50 | .65 | 0 | 1 | .071 | .122 |
| 19 Difficulties Concentrating | 1.13 | .79 | 1 | 1 | .87 | .71 | 1 | 1 | **.008** | **.177** |
| 20 Fatigue | 1.12 | .72 | 1 | 0 | 1.06 | .74 | 1 | 0 | .521 | .043 |
| 21 Loss of Sexual Interest | 1.22 | 1.18 | 1 | 2 | .95 | 1.06 | 1 | 1.5 | .102 | .110 |
|  | Note: group comparison based on Mann-Whitney U Test with r = effect size based on two-sample rank-sum test, MDN = Median, IQR = interquartile range | | | | | | | | | |
