# Supplementary material for: Measuring quality of life with the Parkinson’s Disease Questionnaire-39 in people with cognitive impairment
Source: PLoS One. 2022 Apr 1;17(4):e0266140. doi: 10.1371/journal.pone.0266140 (PMC8975160; doi:10.1371/journal.pone.0266140)
Supplement: S2 Table — (DOCX) [file pone.0266140.s004.docx]

**Supplement Table 2.** Convergent validity of the PDQ-39 for people with varying levels of cognitive impairment.

| **PDQ-39** | **PDD (N = 94)** | | | **MCI (N = 91)** | | | **Normal (N = 36)** | | |
| --- | --- | --- | --- | --- | --- | --- | --- | --- | --- |
|  | **BDI-II** | | | **BDI-II** | | | **BDI-II** | | |
|  | **Rho** | **p** | **CI** | **Rho** | **p** | **CI** | **Rho** | **P** | **CI** |
| PDQ-39 total score | .386 | < .001 | .20, .55 | .437 | < .001 | .25, .59 | .678 | <.001 | .45, .82 |
| Mobility | .265 | .01 | .07, .44 | .181 | .085 | -.03, .34 | .532 | .001 | .25, .73 |
| Activities of Daily Living | .250 | .015 | .05, .43 | .263 | .012 | .06, .45 | .500 | .002 | .21, .71 |
| Emotional Well-Being | .446 | < .001 | .27, .60 | .604 | < .001 | .45, .72 | .719 | <.001 | .51, .85 |
| Stigmatization | .133 | .203 | -.07, .33 | .224 | .033 | .02, .41 | .415 | .012 | .10, .65 |
| Social Support | .400 | < .001 | .22, .56 | .213 | .042 | .01, .40 | .506 | .002 | .21, .72 |
| Cognition | .274 | .008 | .08, .45 | .600 | < .001 | .45, .72 | .690 | <.001 | .47, .84 |
| Communication | .238 | .021 | .04, .42 | .184 | .081 | -.02, .38 | .425 | .010 | .11, .66 |
| Bodily Discomfort | .158 | .129 | -.05, .35 | .292 | .005 | .09, .47 | .304 | .072 | -.03, .58 |
| ***** PDQ-39: Parkinson’s Disease Questionnaire 39; BDI-II: Beck’s Depression Inventory II, MOCA: Montreal Cognitive Assessment, Rho = Spearman’s Rho ρ  PDD: MOCA <21, MCI: MOCA 21-25, Normal: MOCA ≥26 | | | | | | | | | |
