# Supplementary material for: Measuring quality of life with the Parkinson’s Disease Questionnaire-39 in people with cognitive impairment
Source: PLoS One. 2022 Apr 1;17(4):e0266140. doi: 10.1371/journal.pone.0266140 (PMC8975160; doi:10.1371/journal.pone.0266140)
Supplement: S3 Table — (DOCX) [file pone.0266140.s005.docx]

**Supplement Table 3.** Convergent validity of the PDQ-39 and the NMS-Q for people with low and high MOCA score.

| **PDQ-39** | **MOCA < 21** | | | **MOCA ≥ 21** | | |
| --- | --- | --- | --- | --- | --- | --- |
|  | **NMS-Q (N = 89)** | | | **NMS-Q (N = 116)** | | |
|  | **Spearman** | **p** | **CI** | **Spearman** | **p** | **CI** |
| PDQ-39 total score | .488 | < .001 | .32, .63 | .601 | < .001 | .48, .71 |
| Mobility | .249 | .017 | .05, .43 | .387 | < .001 | .22, .53 |
| Activities of Daily Living | .390 | < .001 | .20, .55 | .372 | < .001 | .21, .52 |
| Emotional Well-Being | .264 | .011 | .06, .44 | .655 | < .001 | .54, .75 |
| Stigmatization | .242 | .020 | .04, .43 | .306 | < .001 | .13, .46 |
| Social Support | .296 | .004 | .10, .47 | .291 | .001 | .12, .45 |
| Cognition | .490 | < .001 | .32, .63 | .682 | < .001 | .57, .77 |
| Communication | .363 | < .001 | .17, .53 | .450 | < .001 | .29, .58 |
| Bodily Discomfort | .372 | < .001 | .18, .53 | .509 | < .001 | .36, .63 |
| \| ***** PDQ-39: Parkinson’s Disease Questionnaire 39; NMS-Q: Non-motor symptoms questionnaire, MOCA: Montreal Cognitive Assessment, CI: 95% confidence interval \| \| --- \| | | | | | | |
