# Supplementary material for: Measuring quality of life with the Parkinson’s Disease Questionnaire-39 in people with cognitive impairment
Source: PLoS One. 2022 Apr 1;17(4):e0266140. doi: 10.1371/journal.pone.0266140 (PMC8975160; doi:10.1371/journal.pone.0266140)
Supplement: S4 Table — (DOCX) [file pone.0266140.s006.docx]

**Supplement Table 4.** Convergent validity of the PDQ-39 and the UPDRS for people with low and high MOCA score.

| **PDQ-39** | **MOCA < 21** | | | **MOCA ≥ 21** | | |
| --- | --- | --- | --- | --- | --- | --- |
|  | **UPDRS (N=48)** | | | **UPDRS (N = 60)** | | |
|  | **Spearman** | **p** | **CI** | **Spearman** | **p** | **CI** |
| PDQ-39 total score | .450 | .001 | .19, .65 | .547 | < .001 | .35, .70 |
| Mobility | .461 | .001 | .21, .66 | .630 | < .001 | .45, .76 |
| Activities of Daily Living | .509 | < .001 | .27, .69 | .491 | < .001 | .28, .66 |
| Cognition | .303 | .030 | .02, .54 | .332 | .008 | .09, .54 |
| Bodily Discomfort | .119 | .416 | -.17, .39 | .448 | < .001 | .22, .63 |
| \| Note: PDQ-39: Parkinson’s Disease Questionnaire 39; UPDRS: Movement-Disorder-Society sponsored  unified Parkinson’s Disease Rating Scale, motor assessment, MOCA: Montreal Cognitive Assessment,  CI: 95% confidence interval \| \| --- \| | | | | | | |
